# *Campylomorphus homalisinus* (Elateridae): a new species for Lombardy (Italy), with notes on its ecology, distribution and biogeography

**DOI:** 10.3897/BDJ.2.e1075

**Published:** 2014-03-31

**Authors:** Paolo Biella, Riccardo Groppali

**Affiliations:** † Dept. of Earth and Environmental Sciences, University of Pavia, Pavia, Italy

**Keywords:** Campylomorphus homalisinus, Coleoptera, Flower visitor, Flower Nectar, Apennines, Pleistocene Glaciations, New record

## Abstract

*Campylomorphus homalisinus* has been found on Mt. Lesima (Northern Apennines) and it is the first record for the Lombardy region. *Campylomorphus homalisinus* is a rare orophilous species: it has a discontinuous chorology that may have been caused by glaciers dynamics during the Pleistocene era. Little is known about the ecology of the species. This record and the expert-based investigation we performed determined that *Campylomorphus homalisinus* inhabits shrublands and grasslands, but may also occur in the forests. This survey includes the only record of *Campylomorphus homalisinus* foraging on flowers, a behavior that is not rare in the family Elateridae. We hypothesize that adults integrate their diet with flower resources according to a generalist strategy.

## Introduction

Coleopterans play an important role in pollination (Faegri and van der Pijl 1979) being among the most frequent flower visitors (Gómez et al. 2013). As a matter of fact, Elateridae are often sampled by aspiration on flowers Platia and Akrawi 2013). Due to unspecialized masticatory apparatus (in comparison to others taxa like bees) Coleoptera usually forage on disc and open flowers, where the resources are easily available (Willmer 2011), although it is worth highlighting that they can cause damage to reproductive structures of flowers (Leavitt and Robertson 2006).

As stated by Platia (1994), “a very big amount of work is still to be done in order to study the biology and ethology of the most part of Elaterid species”. One of the less studied species is *Campylomorphus homalisinus* (Illiger, 1807). It was named as *Elater homalisinus* by Illiger (1807) from a specimen from Portugal, but in 1860 Jacquelin du Val considered it as a monospecific genus. Its larval stages and habits remain unknown (Platia 1994). Moreover, the exact distribution of the species is uncertain. At present, online databases provide either poor information or no records at all. Some historic works highlight the presence of the species in Italy, France and Iberian Peninsula (Chevrolat 1840), without giving details. Some ancient records of Deyrolle (in Chevrolat 1840) refers to places by names which are not in use in modern times and therefore are not directly identifiable. Other records are biogeographically unlikely, for example a record at Mt. Rosa (Stierlin 1886). In the case of Mt. Cenis (Deville 1935), the presence of the species has not been confirmed by recent saproxylic insects’ surveys (SAPROX, Inventaire des coléoptères saproxyliques de France métropolitaine, available on inpn.mnhn.fr). Such cases might be explained by incorrect identification (Platia, *in verbis*). It is really important to update the distribution of *Campylomorphus homalisinus*: to improve current knowledge about its ecology and biogeographic history. In addition, changes in species range (expansion or contraction) might reflect response capability to a changing environment, and thus survival possibility. Species ranges and especially their dynamic are so important that the International Union for Conservation of Nature (IUCN 2001) includes two criteria related to species biogeography in the process of assessing the threatened status of species: Extent Of Occurrence (EOO) and Area Of Occupancy (AOO).

We have three questions about *Campylomorphus homalisinus*: 1) Has it ever been collected in the Lombardy region (Italy)? 2) What are its global distribution and altitudinal range? 3) What is currently known about its ecology?

## Materials and methods

To record flower foraging Elateridae a sampling scheme based on three plots of 2,5×2,5 m was established on Mt. Lesima (Pavia, see *Study area*). During the summer 2013 they were sampled weekly for 20 minutes, twice a day. In each sampling day the starting plot was chosen at random. Captures were stored in a 70% ethyl alcohol solution and the foraged plant was recorded. The specimens have been identified by a specialist (Dr. G. Platia).

Historic records of *Campylomorphus homalisinus* have been verified with Museum collections and published works. Two experts (G. Platia and J.-L. Zapata de la Vega) have been interviewed about the ecology and the records of the species.

With the purpose of assessing a possible sub-regional differentiation in the altitudinal range of the species, the frequency of Italian records of *Campylomorphus homalisinus* above 1500 m has been calculated for the portion of its Italian distribution range to the west of Mt. Lesima, and for the portion of the Italian distribution range to the east of the same mountain.

### Study area

**Figure 1.**
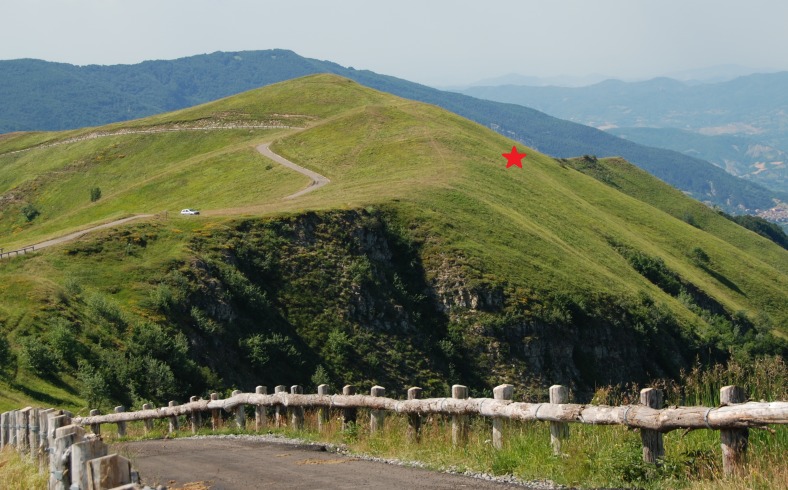
Study area of Mt. Lesima (Northern Apennines, Lombardy region, Italy). The star symbol shows the sampled grassland. Photo by P. Biella.

The collection took place in a montane grassland formation with a north-eastern exposure and average altitude of 1650 m [Fig. 1]. This grassland is situated in the Lombardy side of Mt. Lesima, 44°41'6 N 9°15'26 E (a slope of the mountain is included in Emilia-Romagna region). Mt. Lesima (1724 m a.s.l.) is included into the area southern to the Po river named Oltrepò Pavese, that consists of many hills and some higher mountains of the Northern Apennines. According to Rossetti and Ottone (1980), the average rainfalls of the area that rounds Mt. Lesima is 1250–1500 mm and the mean annual temperatures is 5 °C. In the higher peaks, rainfall level is even greater considering both the orographic-lift effect in rainy clouds formations and the effect of moisture addition to air masses by daily heating, which is more frequent in the summer. The Temperate Oceanic Submediterranean bioclimate is the prevailing climate type in the mountainous area.

Vegetation at lower elevations of Oltrepò Pavese is dominated by forests of Downy Oak (*Quercus pubescens*) and Manna Ash (*Fraxinus ornus*) with *Carex flacca* and *Brachypodium rupestre* in the grassed stages of the vegetational series. Above the altitude of 800 m vegetational stages of the series *Trochiscantho nodiflori* – *Fago sylvaticae sigmetum* are established, which are dominated by extended forests of Beech (*Fagus sylvatica*) (Verde et al. 2010). Some intermediate stages are dominated by mesophilous grasslands of the *Festuco-Brometea* typology, which are encountered mostly above 1550 m (Verde et al. 2010) and they are managed as pastures. On Mt. Lesima about 130 ha of flowered grasslands of *Festuco-Brometea* typology occur on a geological substrate of limestone and marl named “Calcari di Monte Antola”. They are partly colonized by shrubs as *Sorbus aucuparia*, *Rosa* sp., *Genista tinctoria*, *Genista radiata*, *Vaccinium uliginosum* and *Vaccinium myrtillus*.

## Taxon treatments

### Campylomorphus homalisinus

Illiger, 1807

#### Materials

- **Type status:** Other material. **Occurrence:** recordedBy: Paolo Biella; individualCount: 1; sex: Male; **Location:** country: Italy; stateProvince: Lombardy, Pavia province; verbatimLocality: mt. Lesima; verbatimElevation: 1650; verbatimLatitude: 44° 41.103'N; verbatimLongitude: 9° 15.443'E; **Identification:** identifiedBy: G. Platia; dateIdentified: 09-2013; **Event:** samplingProtocol: 3 plots of 2,5×2,5 mt, jar with 70% ethyl alcohol; eventDate: Summer 2013

## Discussion

**Figure 2.**
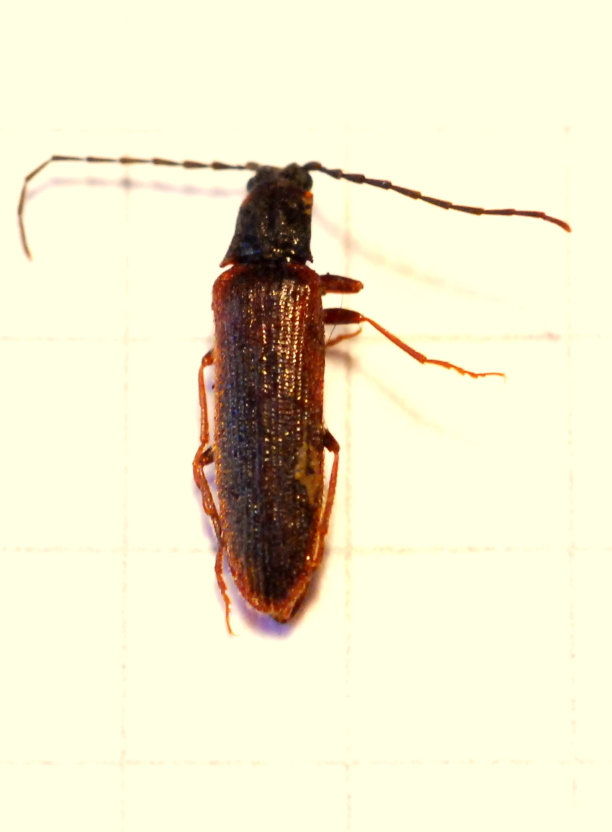
*Campylomorphus homalisinus* of the picture was collected foraging on flowers of *Laserpitium siler* (*Apiaceae*). Photo by P. Biella.

Data collected on Mt. Lesima yielded the first record of *Campylomorphus homalisinus* (Illiger, 1807) inside Lombardy (Italy) [Fig. 2]. A male specimen was recorded foraging on a flower (*Laserpitium siler*, Apiaceae): according to the literature, a flower foraging *Campylomorphus homalisinus* has never been recorded before this sampling.

### Biogeography of *Campylomorphus homalisinus*

It is not known whether this species has always been present in the area, but it is certain that it has not been detected in many surveys set in Lombardy (Ruffo and Stoch 2005, Mazzoldi 1982, Villa and Villa 1844, Cavagna Sangiuliani Di Gualdana et al. 1864). Roberti et al. (1965) did not record it in their surveys, which cover some peaks of Oltrepò Pavese, the area where Mt. Lesima is located. Additionally, records for Lombardy of this species are not present in the entomological collections of three Italian Natural History Museums: Natural History Museum of Milano, Natural History Museum of Genova and Natural History Museum of the University of Pavia. The hypothesis of an expansion northwards of the occupancy area should not be rejected. Indeed, 4 specimens of the species were collected in the nearby Mt. Antola (Genova province, Liguria, stored in the collection of Natural History Museum of the University of Pavia, dated 1919 and 1939), which is only 15 km from Mt. Lesima and has similar altitude and habitats.

**Figure 3.**
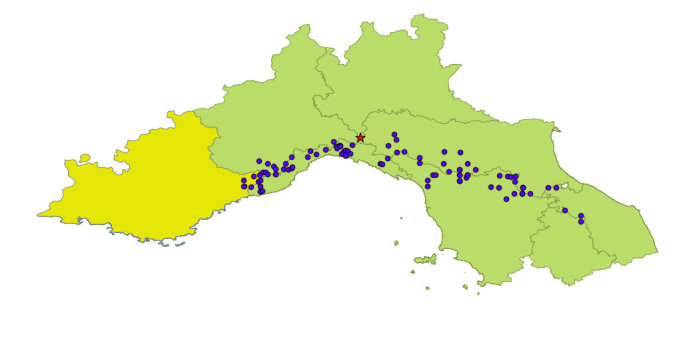
Eastern distribution area of *Campylomorphus homalisinus*. Light green signs Italian administrative regions, yellow is the French one. Blue points sign occurrences of *Campylomorphus homalisinus* according to literature, databases and museums collections. The red star shows mt. Lesima record. Administrative province of occurrence are Provence-Alpes-Côte d'Azur (France) and Cuneo, Imperia, Savona, Genova, La Spezia, Pavia, Piacenza, Parma, Reggio nell'Emilia, Modena, Bologna, Lucca, Massa-Carrara, Pistoia, Firenze, Forlì-Cesena, Arezzo, Pesaro-Urbino, Perugia (Italy).

**Figure 4.**
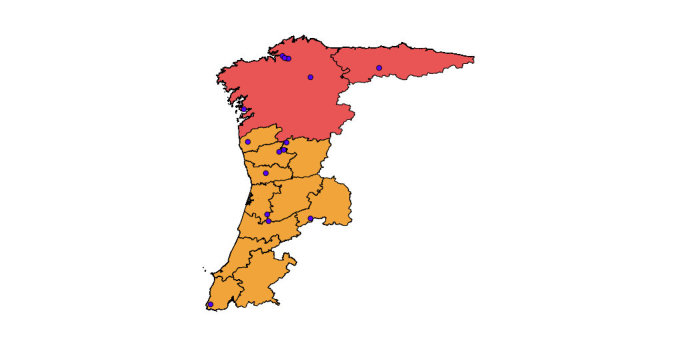
Western distribution area of *Campylomorphus homalisinus*. Red colour signs administrative regions of Spain, Portuguese ones are in orange. Blue points sign occurrences of *Campylomorphus homalisinus* according to literature, databases and museums collections. Administrative provinces of occurrence are Oviedo, Lugo, A Coruña, Orense, Pontevedra (Spain) and Viana do Castelo, Braga, Vila Real, Lisboa, Guarda, Coimbra, Aveiro (Portugal).

With regards to the biogeography of *Campylomorphus homalisinus*, it is an orophilous European click beetle that occurs in the Iberian Peninsula (Zapata de la Vega and Sánchez-Ruiz 2012, Sánchez-Ruiz 1996) [Fig. 4], France and Italy (Platia 1994) [Fig. 3]. The distance between the most western record in France and the most eastern record in Spain is almost 10^3^ km in straight line (computed with GoogleEarth). This lack of evidence for occurrence is reliable, because many exhaustive surveys covering most part of France did not record it outside the Mercantour National Park (Callot 1990, Therond 1975, Houlbert 1921, SAPROX: *Inventaire des coléoptères saproxyliques de France métropolitaine* which data are available on inpn.mnhn.fr).

How can this discontinuous distribution be explained? During the Pleistocene era, ice-sheets extended their surface covering Central Europe several times. During glaciers expansions, species migrated towards southern not-iced refugia, namely the Iberian, Italian and Balkan Peninsulas. During Earth's warmer periods the glaciers receded and some species colonized the newly uncovered land. Some of them established continuous areas, like the mammal *Erinaceus europeus* (Randi 2007) or the grasshopper *Chorthippus parallelus* (Lunt et al. 1998), others underwent speciation and generated either endemisms like many small mammal species (Amori et al. 1996) or subspecies like the Scots pine in the Iberian Peninsula (Gómez and Lunt 2007). In Italy, Pedroni (2007) recorded a high proportion of Turanic species in the Northern Apennines Elaterid fauna and explained that as result of glaciers dynamics. Might the discontinuous distribution of *Campylomorphus homalisinus* be explained in such a scenario? A continuous area of occurrence was probably present before the species took refuge in two different localized areas: the Iberian Peninsula and the Italian one. However, after the glaciers receded, the species failed to occupy its previous area. In fact, it is believed that colonization of central Europe post glaciations has been carried from extra-Mediterranean refugia because mountainous barriers like Pyrenees and Alps isolated the Mediterranean ones (Randi 2007). This may be a reason why *Campylomorphus homalisinus* could not establish a continuous area after ice-sheet recession, having only taken refuge in the Mediterranean area. Is it still the same species in both peninsulas? Dr. Platia states that specimens of the two distributive areas are morphologically identical (Dr. Platia, *in verbis*) but a genetic analysis could help in validate such observation.

### Ecology

In Italy and France, *Campylomorphus homalisinus* is orophilous, being present at altitudes between 400 m and 1900 m (CK-map database in Ruffo and Stoch 2005). In the Iberian Peninsula, such range is extended towards lower altitudes: 50 m a.s.l. (Baselga and Novoa 2004). Thus, the altitudinal range differs between the two parts of the species' range. Furthermore, focusing on the Italian sector, there is a tendency to occupy higher altitudes in the western area (40% of records are above 1500 mt), while in the eastern only 5% of records are above 1500 mt.

**Figure 5.**
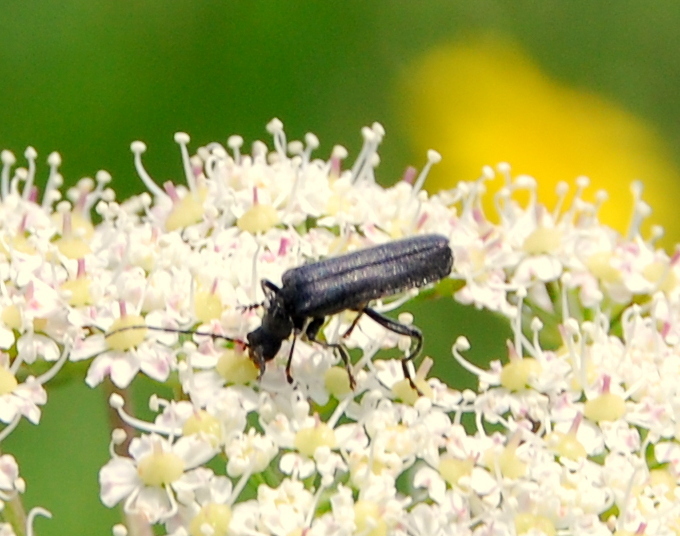
*Limonius minutus* (Linné, 1758) feeding nectar: it is obvious the contact between the mouthparts and the exposed nectaries of *Lasepitium siler* (Apiaceae). Photo by P. Biella.

Fauna d’Italia (Platia 1994) hypotesized that the habitat of the species may be deciduous forests and clearings. Our survey detected it on a flowered grassland of 130 ha. *Campylomorphus homalisinus* has been found there together with *Athous flavipennis* (Candèze, 1863) and *Limonius minutus* (Linné, 1758) (Table 1). In the Iberian Peninsula, records of *Campylomorphus homalisinus* are associated to Elateridae that frequent grasslands and shrublands like *matorral* (Zapata de la Vega, *in verbis*). In Italy, Platia once recorded *Campylomorphus homalisinus* on the grassed top at a altitude of 1700 m a.s.l. (Mt. Catria, Platia *in verbis*) without any trees. This situation is very similar to the studied area. Furthemore, on Mt. Lesima the captured species were feeding on nectar (Fig. 5), and also *Campylomorphus homalisinus* was captured during nectar feeding: thus the species uses grassland resources. Despite little available information, our observations on the ecology of *Campylomorphus homalisinus* suggest that clearings and grasslands are inhabited by the species, without excluding the presence in forests. Data on the larval habitat are not available but it is possible that the larva inhabits forests, as it may live in the dead wood or underground humus (Platia, *in verbis*). Finally, the female is very difficult to record (9 females out of 96 specimens have been collocated in G. Platia’s personal collection, Platia *in verbis*), so it may live hidden and come out very rarely.

In the Ck-map database (Ruffo and Stoch 2005) records of 22 species of Elateridae are present in 7 mountainous localities near the sampled grasslands (Mt. Lesima, Mt. Ebro, Montecapraro, Brallo di Pregola, Bobbio, Mt. Penice, Mt. Antola). According to Fauna d’Italia (Platia 1994), 10 of these species feed on flowers: 4 are species inhabiting clearings and forest edges, 6 live in grasslands. Six others species inhabit either grasslands or clearings; also in this cases the usage of flowering plants resources is possible. Thus, flower feeding is quite widespread in the Elateridae of this area: 45% of Elateridae species of this area feed on flowers, with the percentage rising to 68% when possible additional cases are considered. It is a surprisingly higher precentage than other areas: 22% of the species in Valle d'Aosta (Pedroni and Platia 2002). As highlighted by this sampling, by the other Elateridae recorded in the area and according to the literature (Arnett et al. 2002, Costa et al. 2010, Mulerčikas et al. 2012) we argue that diet integration with flower rewards might be crucial for adult Elateridae. In fact, pollination services are documented: species of the family had already been found on the generalist plant *Erysimum mediohispanicum* (Gómez et al. 2013), on the generalist *Ilex opaca* shrub (Rathcke 1988) and on tropical plants (Irvine and Armstrong 1990). The two plant species from which Elateridae were collected on Mt. Lesima have open flowers, but they differ in colour, presence of short tube and reward accessibility: *Laserpitium siler*, which *Campylomorphus homalisinus* and *Limonius minutus* feed on, has disc white flowers with accessible nectar, grouped in umbrella-shape blooms; *Knautia drymeia*, which *Athous flavipennis* feed on, has pink short tubular flowers grouped in head-blooms. Thus, the records on flowers of different morphology and the case, among others, of orchids pollinated by Elateridae (Kullenberg 1961) hint that the family Elateridae’s foraging activity on flowers is not bound by selective plant traits. In the case of Elateridae resource usage of Mt. Lesima is occurring a functional generalization (*sensu* Ollerton et al. 2007) which leads them to be opportunistic and to depend less specifically on constrained or poorly abundant specific resources: being generalists a higher resource uptake is guaranteed in a traveling animal (Waser et al. 1996), and indeed travel is energy-consuming.

## Supplementary Material

XML Treatment for Campylomorphus homalisinus

## Figures and Tables

**Table 1.** Elateridae of Mt. Lesima (Italy, Lombardy, PV) – Notes on ecology from Platia (1994) are marked with "×". "+" denotes data from our observations.

Mt. Lesima Elateridae	Chorology	Orophilous	Forests	Clearings	Shrubs	Grasslands	Flowers
Athous (Haplathous) flavipennis (Candèze, 1863)	South Europe	×	×				
*Campylomorphus homalisinus* (Illiger, 1807)	South Europe, discontinous	×	×	×		**+**	**+**
*Limonius minutus* (Linné, 1758)	Eurosibiric	×	×	×	×		×
